# Aneurysmal bone cyst of the mandible with conservative surgical management: A case report 

**DOI:** 10.4317/jced.55771

**Published:** 2019-06-01

**Authors:** Efraín-del Cristo Álvarez-Martínez, Mónica-Vanessa Posso-Zapata, Vanessa-Andrea Flórez-Arango, Johan-Sebastián Lopera-Valle, Carlos-Martín Ardila

**Affiliations:** 1 Oral and maxillofacial surgeon. University of Antioquia. Medellín, Colombia; 2 Postgraduate student of Oral and Maxillofacial Surgery. University of Antioquia. Medellín, Colombia; 3 Postgraduate student of Radiology. University of Antioquia. Medellín, Colombia; 4 Periodontist, Ph.D. in Epidemiology, Coordinator of the Biomedical Stomatology Research Group. Medellín, Colombia

## Abstract

The aneurysmal bone cyst is a nonneoplastic, osteolytic and locally destructive lesion that mainly affects the metaphyseal area of long bones and only 2% of it is diagnosed in the maxillofacial skeleton. Although surgical treatment is the most common option, it is associated to high morbidity rates. The case of an aneurysmal bone cyst of a considerable size in a 27-year-old male patient illustrating a conservative surgical approach with preservation of the dental structures in the mandible to limit aesthetic and functional side effects is presented. Two-year clinical follow-up was performed with no evidence of recurrence.

** Key words:**Aneurysmal bone cyst; curettage, conservative treatment, mandibular osteotomy.

## Introduction

The aneurysmal bone cyst (ABC) is a benign intraosseous lesion that develops in patients under 30 years, its location is usually the metaphysis of long bones and only 2% of cases are diagnosed in the maxillofacial skeleton; it represents 1,5% of nonodontogenic maxillary cysts, and has a 3:1 mandible/maxilla rate (especially in the posterior area) (1).

The ABC is generally diagnosed in young people between their twenties and thirties, has no predilection for any gender, usually affects long bones (50%), vertebral column (20%), and around 2% manifests in the bones of the mandible (2,3). According to the World Health Organization, this lesion is classified within the giant-cell lesions, together with central and peripheral giant-cell granuloma, cherubism and simple bone cyst (4).

ABC is a rare, expansive, locally destructive lesion and it constitutes a diagnostic and therapeutic challenge in the daily clinical practice (2). Therapeutic management for ABC is still controversial; the approach depends on factors such as age, location, extent and size of the lesion. Several treatment options, such as simple curettage, en bloc resection, radiation therapy, embolization or a combination of these methods, have been proposed.

Although surgical treatment is the most common alternative, it is associated to high morbidity rates including postoperative aesthetic and functional alterations, usually as a consequence of extensive resections, in expansive lesions. This article presents the case of a mandibular ABC of significant size successfully managed through a conservative surgical approach aimed at limiting the aesthetic and functional side effects in a young patient.

## Case Report

**Figure 1:**
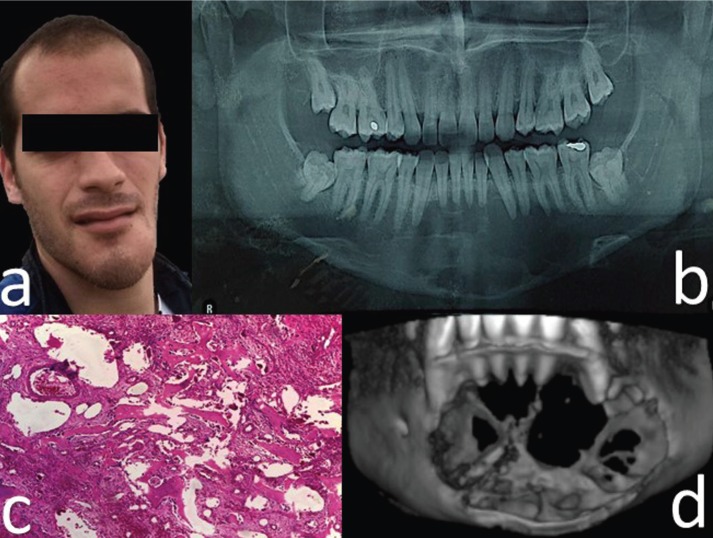
a. Pre-surgical evaluation with evident asymmetry of the lower third, mainly the left side. b. Pre-treatment panoramic radiograph with multilocular radiolucent lesion showing irregular edges and extending from the mesial root of tooth #36 to #44 with compromised interradicular bone. Note the root resorption of teeth #31, #41 and #42. c. Histopathology study (Hematoxylin Eosin) with blood-filled vesicular channels surrounded by lamellar bone, with spicules surrounded in turn by osteoblasts. Multinucleated giant cells and abundant fibroblasts are also described. d. 3D reconstruction of front-view computed tomography showing 29 x 45 x 30 mm expansive lithic lesion related to the perforation of the vestibular and lingual bone tables.

27-year-old male patient with no underlying systemic diseases who sought medical attention because of a three-month medical case of facial asymmetry from increased volume of the left chin area (Fig. 1a), with no other associated symptoms. The patient claimed he had been subject to facial trauma in the same area two years before.

Adequate mouth opening and mandibular mobility were found in the clinical examination; no reactive lymphadenopathies were found. Permanent dentition, stable occlusion and displacement and rotation of tooth #33 were observed in the intraoral examination; swelling of the soft tissues and effacement of the bottom of the sulcus between teeth #34 and #42 were found. The oral mucosa was found to be healthy, smooth, firm and with no pain during palpation.

Panoramic radiography revealed a multilocular radiolucent lesion with no defined borders, extending from the mesial root of tooth #36 to tooth #44, that affected the interradicular bone, with root resorption of teeth #31, #41 and #42 (Fig. 1b).

With a diagnostic impression of ameloblastoma versus central giant-cell granuloma, a biopsy was taken under local anesthesia after 4cc aspiration of dark hematic secretion. Histopathological analysis confirmed the ABC diagnostic; blood-filled vesicular channels surrounded by lamellar bone with spicules surrounded in turn by osteoblasts were described. Multinucleated giant cells and abundant fibroblasts were also observed (Fig. 1c). Additionally, the CT scan evidenced this expansive lytic lesion of 29 x 45 x 30 mm associated to a perforation of the vestibular and lingual bone tables (Fig. 1d).

Considering the patient’s age, the dimensions of the lesion and the potential functional and aesthetic consequences of the en bloc resection, bone curettage and additional endodontic treatment of the anteroinferior teeth was performed, anticipating the compromise of the vascular and nervous contribution due to the extension of the lesion to the apex of these teeth; Likewise, a semi-rigid wire and resin splint was used to stabilize the dentoalveolar segment.

**Figure 2:**
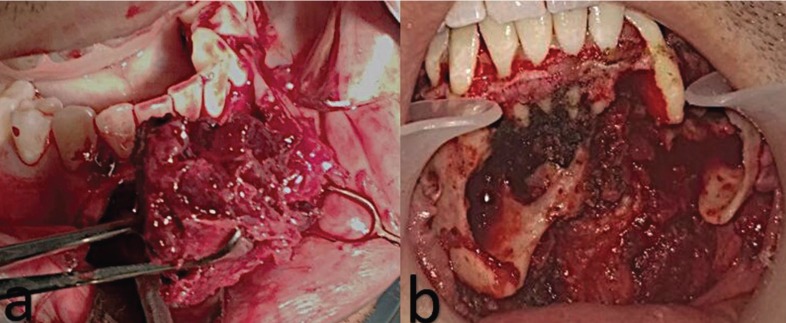
a. Aneurysmal bone cyst removal. b Surgical site with preservation of dental structures.

A circumvestibular surgical incision of approximately 34 to 43 was performed under general anesthesia to gain access to the lesion; a highly vascularized lesion with evident angiogenesis was observed; profuse bleeding was controlled through local procedures and cauterization. Once the tumor was removed, the surgical site, including the basilar rim, was extended 5mm; to stabilize the mandible, two 2.7 mm preformed reconstruction plates associated to the filling of the defect with bone graft were placed. Flaps were repositioned and the procedure finished with no immediate complications (Fig. 2). While maintaining a stable occlusion, feeding was achieved via nasogastric tube for 15 days.

**Figure 3:**
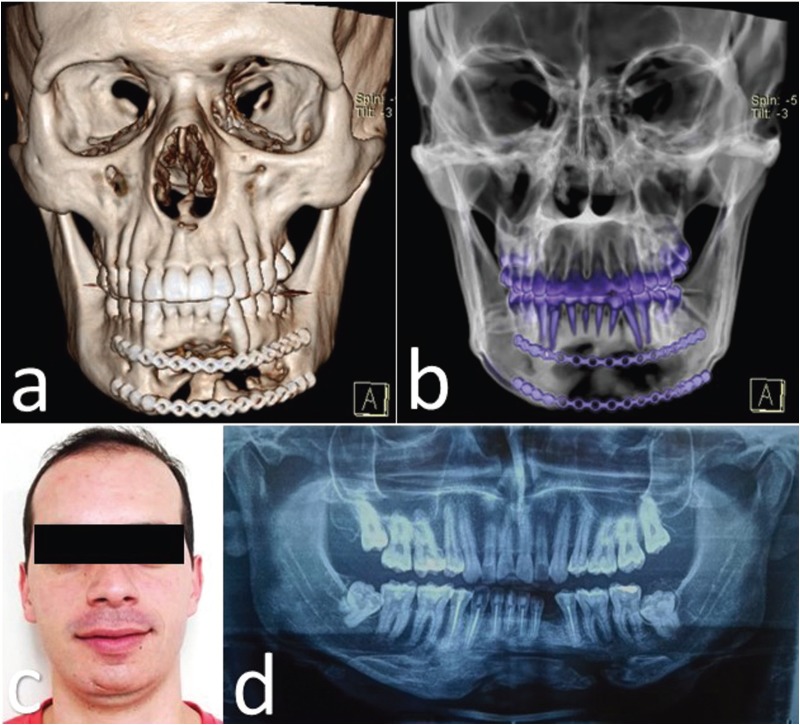
a,b. 3D reconstruction of front-view computed tomography 6 months after the postoperative check-up showing adequately positioned titanium plates and ongoing bone remodeling. c. Twenty-four months of postoperative follow-up, with no facial asymmetry. d. Panoramic radiograph after 24 months of postoperative follow-up showing bone defect in the surgical site associated with corticalization of the edges and absence of residual lesions.

After two months of postoperative follow-up, an 8mm dehiscence with root exposure of tooth #33 was observed in the vestibular area, so it was extracted considering its poor prognosis. The patient was evaluated through tomographies (Fig. 3a,b), clinically (Fig. 3c) and radiographically (Fig. 3d) for two years, no tumoral recurrence was identified during that time; aesthetic and functional results were satisfactory. A written consent of the patient according to ethical principles was signed.

## Discussion

The etiology and pathogenesis of ABC is not very clear. The most accepted theory is the existence of previous trauma (5,6), which corresponds to what the patient reported in this case. Likewise, as described in the literature, it also develops in young patients (2,3).

In this case, correspondence with the literature was also observed, as the ABC usually develops as an expansive, well-defined, uni or multilocular lytic lesion with thin sclerotic margins, liquid-liquid levels, as well as different degrees of cortical rupture (Fig. 1d) and extension to adjacent soft tissues (6).

Histological characteristics are related to the replacement of bone with fibro-osseous tissue and multinucleated giant cells, with blood-filled sinusoidal or cavernous spaces (2,3,4,7), as observed in this case (Fig. 1c).

Considering the differential diagnosis, it is important to bear in mind that ABCs usually expand to a greater degree and are more frequent in the posterior aspect of the mandible than giant-cell granulomas, while ameloblastoma is more frequent in older patients, and cherubism is a bilateral multifocal disease (8).

The treatment for ABC depends on the age, extension, aggressiveness, size and location of the lesions; It ranges from simple curettage to extensive resection with subsequent rehabilitation (9-12). Most ABCs are associated with another pathological entity such as ossifying fibroma, central giant-cell granuloma or benign osteoblastoma, which conditions the aggressiveness of the treatment to be considered (1). Cottalorda and Bourelle (13) suggested that inactive lesions can only be cured with biopsy or curettage; however, in active or aggressive lesions, resection offers a satisfactory theoretical solution (14).

One of the therapeutic challenges of the ABC aimed at reducing postoperative morbidity involves avoiding damage to the alveolar neurovascular bundle included or adjacent to the neoplastic lesion. Similarly, rigorous resection or curettage in all cyst walls should be oriented towards preserving as many dental pieces as possible, without considerably increasing the risk of recurrence (15).

In this case, considering the patient’s age, the compromised area and future oral and facial rehabilitation, a conservative surgical management was chosen. The treatment with bone curettage, in combination with the performed endodontics had a positive response, the lesion healed and an adequate facial aesthetic with preservation of masticatory function and phonation was achieved. In this case, an en bloc resection with anteroinferior dental loss that would have required more complex instances of rehabilitation due to the high degree of aesthetic and functional consequences was avoided.

The highest ABC recurrence rates (21%) (10) have been reported in relation to bone curettage due to the persistence of residual lesion, considering the absence of well-defined edges or capsule at the time of the procedure (11,12). In this case, no recurrence was observed during the follow-up of two years, which is the time in which most scientific evidence reports it (11-15)
